# Cardiovascular effects in patrol officers are associated with fine particulate matter from brake wear and engine emissions

**DOI:** 10.1186/1743-8977-1-2

**Published:** 2004-12-09

**Authors:** Michael Riediker, Robert B Devlin, Thomas R Griggs, Margaret C Herbst, Philip A Bromberg, Ronald W Williams, Wayne E Cascio

**Affiliations:** 1 Institute of Occupational Health Sciences, Rue du Bugnon 19, 1005 Lausanne, Switzerland; 2 U.S. EPA, ORD, National Health and Environmental Effects Research Laboratories, Research Triangle Park, NC, USA; 3 Division of Cardiology, School of Medicine, University of North Carolina, Chapel Hill, NC, USA; 4 North Carolina State Highway Patrol, Raleigh, NC, USA; 5 Center for Environmental Medicine, Asthma and Lung Biology, School of Medicine, University of North Carolina, Chapel Hill, NC, USA; 6 U.S. EPA, ORD, National Exposure Research Laboratories, Research Triangle Park, NC, USA; 7 Brody School of Medicine, East Carolina University, Greenville, NC, USA

## Abstract

**Background:**

Exposure to fine particulate matter air pollutants (PM_2.5_) affects heart rate variability parameters, and levels of serum proteins associated with inflammation, hemostasis and thrombosis. This study investigated sources potentially responsible for cardiovascular and hematological effects in highway patrol troopers.

**Results:**

Nine healthy young non-smoking male troopers working from 3 PM to midnight were studied on four consecutive days during their shift and the following night. Sources of in-vehicle PM_2.5 _were identified with variance-maximizing rotational principal factor analysis of PM_2.5_-components and associated pollutants. Two source models were calculated. Sources of in-vehicle PM_2.5 _identified were 1) crustal material, 2) wear of steel automotive components, 3) gasoline combustion, 4) speed-changing traffic with engine emissions and brake wear. In one model, sources 1 and 2 collapsed to a single source. Source factors scores were compared to cardiac and blood parameters measured ten and fifteen hours, respectively, after each shift. The "speed-change" factor was significantly associated with mean heart cycle length (MCL, +7% per standard deviation increase in the factor score), heart rate variability (+16%), supraventricular ectopic beats (+39%), % neutrophils (+7%), % lymphocytes (-10%), red blood cell volume MCV (+1%), von Willebrand Factor (+9%), blood urea nitrogen (+7%), and protein C (-11%). The "crustal" factor (but not the "collapsed" source) was associated with MCL (+3%) and serum uric acid concentrations (+5%). Controlling for potential confounders had little influence on the effect estimates.

**Conclusion:**

PM_2.5 _originating from speed-changing traffic modulates the autonomic control of the heart rhythm, increases the frequency of premature supraventricular beats and elicits pro-inflammatory and pro-thrombotic responses in healthy young men.

## Background

Exposure to fine particulate matter (PM_2.5_) in the ambient air increases daily deaths [1] and hospitalization for cardiovascular diseases [2] in the U.S. and throughout the world [3] with most effects within one day after exposure. It is estimated that 800,000 excess deaths worldwide each year may be attributable to particulate matter air pollution [4], possibly secondary to myocardial infarction [5], life-threatening arrhythmias [6] or heart failure, as reviewed in a recent American Heart Association scientific statement [7]. Yet, the underlying pathophysiological mechanisms that link PM_2.5 _and cardiopulmonary mortality are poorly understood.

Particles of motor vehicle origin appear to be especially potent with regard to increased mortality [8,9] and hospital admissions due to cardiovascular diseases [10]. Vehicles represent a microenvironment with potentially high exposure to air pollutants from mobile sources. We previously showed that occupational in-vehicle PM_2.5 _exposure to North Carolina Highway Patrol troopers was associated with changes in cardiac parameters, blood proteins associated with inflammation, hemostasis and thrombosis, and increased red blood cell volume (MCV) 10 to 15 hours after completing their shift [11]. These findings were little affected by potential confounders. Controlling for estimates of occupational stress even slightly improved the strength of association with some cardiac parameters. In this paper, we investigated how these health endpoints were associated with specific sources of PM_2.5_.

## Results

### Subjects

Data from nine male non-smoking troopers (8 Caucasian, 1 African-American) were used for the analysis: ten participated, one was excluded due to very high numbers of ectopic beats and high serum cholesterol. This left a total of 36 person-days with valid health data. Their age ranged from 23 to 30 years (mean 27.3 years), their weight from 74 to 102 kg (87 kg), their height from 168 to 191 cm (179 cm), and their body mass index from 24 to 31 kg/m^2 ^(27 kg/m^2^). All were in excellent physical condition.

### Exposure inside the cars and source identification

Elemental PM_2.5_-components and co-pollutants that were correlated to the PM_2.5 _measurements were included in the analysis, if they had over 75% of the data above the reporting limit. Table 1 shows their in-vehicle concentrations and the correlations to the PM_2.5 _measurements. Data of 36 individual samples were available after correction of one silicon-outlier, and replacement of three missing benzene and two missing aldehydes values by their respective means. All concentrations measured were below current occupational threshold limits.

**Table 1:** Components included in the analysis In-vehicle concentrations of the elemental components of PM_2.5 _and gaseous co-pollutants included in the analysis (n = 36 samples): Arithmetic average, standard deviation and correlation (Spearman-Rho) to PM_2.5Mass _and PM_2.5Lightscatter_. *) p < 0.05.

Component	Average	Standard Deviation	Correlation to PM_2.5Mass_	Correlation to PM_2.5Lightscatter_
Benzene (ppb)	3.73	2.9	0.50 *	0.31 *
Aldehydes (μg/m^3^)	34.6	14.9	0.34 *	0.52 *
CO (ppm)	2.6	1.1	0.52 *	0.52 *
Aluminum (Al, ng/m^3^)	66.0	54.5	0.58 *	0.31 *
Silicon (Si, ng/m^3^)	240.0	542.0	0.66 *	0.23
Sulfur (S, ng/m^3^)	1703.0	812.0	0.58 *	0.88 *
Calcium (Ca, ng/m^3^)	48.2	33.5	0.37 *	0.22
Titanium (Ti, ng/m^3^)	11.7	10.0	0.41 *	0.15
Chromium (Cr, ng/m^3^)	2.1	1.7	0.51 *	0.32 *
Iron (Fe, ng/m^3^)	371.0	352.0	0.71 *	0.33 *
Copper (Cu, ng/m^3^)	33.1	18.8	0.16	0.50 *
Selenium (Se, ng/m^3^)	12.6	1.2	0.38 *	0.26
Tungsten (W, ng/m^3^)	5.6	5.9	0.37 *	0.39 *
PM_2.5Mass _(μg/m^3^)	23.0	8.0	1	0.71 *
PM_2.5Lightscatter _(μg/m^3^)	24.1	13.5	0.71 *	1

**Figure 1:**
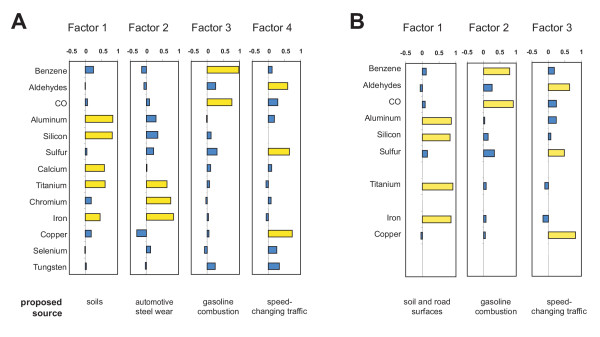
**Source factors loadings. **Factor loadings of the different components of the two models and the proposed sources for these factors. Loadings large than 0.4 are highlighted in yellow.

Source model A consisted of a factor analysis using all elements and co-pollutants listed in Table 1. Four source factors were identified. Figure 1A shows the factor loadings (loadings larger than 0.4 are highlighted) and Table 2 shows the model characteristics. Factor 1 was dominated by silicon and aluminum (named "crustal" factor), factor 2 by iron, chromium and titanium ("steel wear" factor), factor 3 by benzene and carbon monoxide ("gasoline" factor) and Factor 4 by copper, sulfur and aldehydes ("speed-change" factor).

**Table 2:** Source model characteristics Characteristics of the two models and their factors of the principal factor analysis and their associations with the two PM_2.5_-measures: Model A includes all components shown in Table 1, Model B excludes Ca, Cr, Se and W.

Model characteristics	Model A				Model B		
Number of exposure variables included	13				9		
Total variance explained	62.3%				68.9%		
Correlation with PM_2.5Mass _(R^2^)	0.73				0.63		
Correlation with PM_2.5Lightscatter _(R^2^)	0.71				0.52		
Factor characteristics	Factor 1 "crustal"	Factor 2 "steel wear"	Factor 3 "gasoline"	Factor 4 "speed-change"	Factor 1 "road surface"	Factor 2 "gasoline"	Factor 3 "speed-change"
Sum of squares of factor loadings	2.48	2.11	1.82	1.67	3.01	1.68	1.51
Proportion of total variance	19.1%	16.3%	14.0%	12.9%	33.5%	18.6%	16.8%
Slope^a ^of correlation with PM_2.5Mass_	2.95 p = 0.0006	4.11 p < 0.0001	2.16 p = 0.003	4.50 p < 0.0001	4.74 p < 0.0001	3.03 p = 0.002	3.21 p = 0.002
							
Slope^a ^of correlation with PM_2.5Lightscatter_	0.68 p = 0.6 (n.s.)	3.58 p = 0.01	3.74 p = 0.004	11.32 p < 0.0001	2.48 p = 0.15 (n.s.)	4.03 p = 0.026	9.27 p < 0.0001
							

a) Slope in μg/m^3 ^per 1 SD change in the exposure factor score

Source model B was calculated using only elements that were clearly correlated to PM_2.5 _and with the majority of data more than 3 sigma above background noise (i.e., without Ca, Cr, Se and W). Three source factors were identified (Figure 1B and Table 2). Factor 1 was dominated by silicon, aluminum, titanium and iron (named "road surface"); factor 2 by benzene and carbon monoxide ("gasoline"); and factor 3 by copper, sulfur and aldehydes ("speed-change").

The factor "road surface" of Model B was significantly correlated to the factors "crustal" and "steel wear" of Model A (R = 0.80 and 0.64, respectively). Factor "gasoline" of A was correlated to factor "gasoline" of B (R = 0.80); and factor "speed-change" of A to "speed-change" of B (R = 0.91). In contrast, the source factors within each model were completely uncorrelated (R < 0.09).

### Health endpoints associated with sources

**Figure 2:**
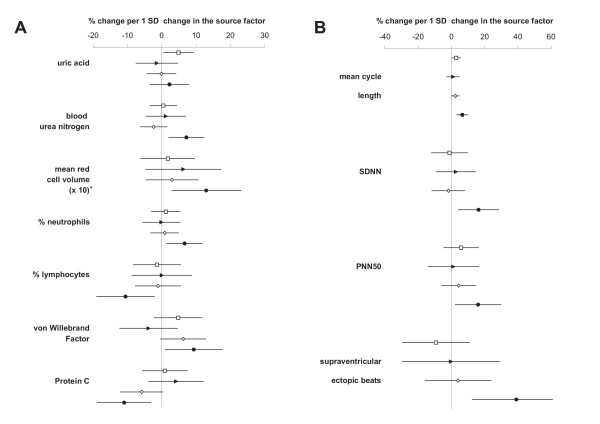
**Associations between Model A and selected health endpoints. **Effect estimates are shown as percent change per one standard deviation change in the source factors. Lines indicate the 95% confidence interval. Symbols represent the different factors: rectangle = "crustal", triangle = "steel wear", diamond = "gasoline" and circle = "speed-change". **Fig 2A: **blood endpoints. *) The estimates for MCV were multiplied by ten to better fit the scale. **Fig 2B: **cardiac endpoints.

**Figure 3:**
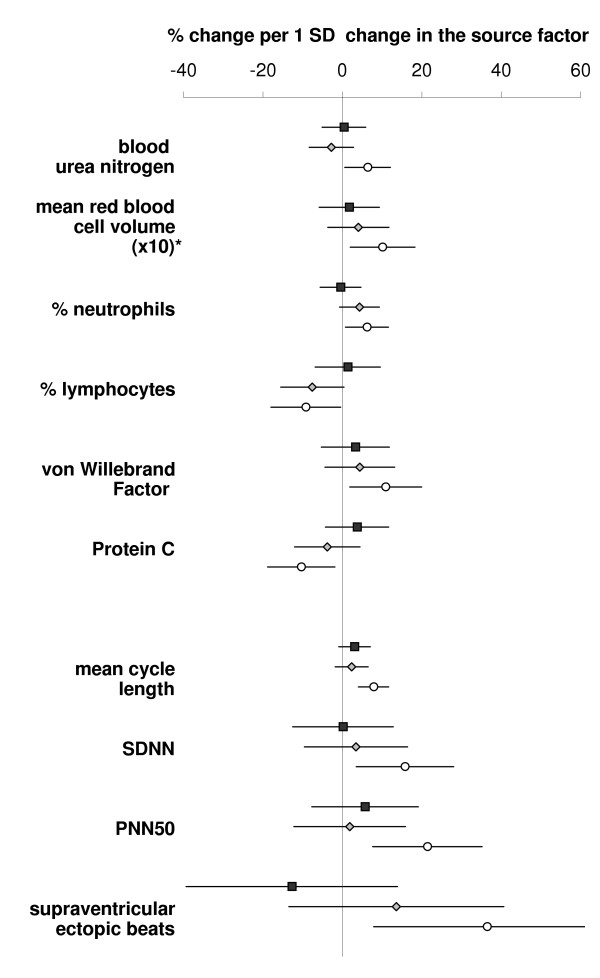
**Associations between Model B and selected health endpoints. **Effect estimates are shown as percent change per one standard deviation change in the source factors. Lines indicate the 95% confidence interval. Symbols represent the different factors: rectangle = "road surface", diamond = "gasoline" and circle = "speed-change". *) The estimates for MCV were multiplied by ten to better fit the scale.

The associations between health endpoints and source factors were studied in a multivariate approach. Figure 2 shows the results for Model A; Figure 3 those for Model B (only health endpoints associated to one of the sources with p < 0.05 are displayed). In both models, most of the significant health effect estimates were associated with the "speed-change" factor (MCL, SDNN, PNN50, supraventricular ectopic beats, % neutrophils, % lymphocytes, MCV, von Willebrand Factor, and protein C). The association with MCV remained unchanged when controlled for osmolality. Two significant associations were observed for the "crustal" factor of Model A (uric acid and MCL), none for the "steel wear" and the "gasoline" factor of either model.

Strong heteroscedasticity (i.e., an indication for a violation of the underlying statistical assumptions) was evidenced in the residual analysis of the models for red blood cell count, hematocrit and hemoglobin (which were significantly associated with the "speed-change" factor). However, every attempt to remove the heteroscedasticity by adjusting the variance-covariance structure also completely removed the significance of these associations.

### Control for potential confounders

The associations observed between factors and health parameters were tested for the following potential confounders: Temperature, relative humidity, the number of law-enforcement activities during the shift (as estimate of workload) and the average speed during the shift. Controlling for these confounders had no visible effect on most effect estimates of the "crustal" and the "speed-change" factor, and the associated health parameters. However, in Models A and B, including these confounders altered the effect estimates with blood urea nitrogen and vWF, especially including all confounders together into the models lowered the effect estimates for the source factor "speed-change" by about one fifth and the confidence interval included zero. In Model A, the estimate for PNN50 was not altered by any of the confounders, but including all confounders into the same model widened the confidence interval to include zero.

## Discussion

We previously reported that in-vehicle exposure to PM_2.5 _was associated with increases in markers of inflammation and coagulation, and modulations of heart rate variability in Highway Patrol troopers [11]. Here we demonstrate that most health endpoints were associated to a PM_2.5 _source factor that reflects speed-changing traffic conditions (dominated by copper, aldehydes and sulfur). Under such driving conditions, copper reflects wear of brakes, aldehydes reflect emissions from accelerating vehicles and sulfur reflects secondary aerosols and possibly diesel combustion products.

In Model A, four principal factors of PM_2.5_-exposure inside the patrol cars were identified. Their loadings suggest the main in-vehicle sources of PM_2.5_. Factor 1 reflects exposure to crustal material from the soils in the study region and the road surface ("crustal" factor). Factor 2 represents wear and tear of mechanical automotive parts, mostly chrome-titanium steels ("steel wear" factor). Factor 3 represents components derived from gasoline combustion ("gasoline factor"). Finally, factor 4 is characterized by components expected from speed-changing traffic ("speed-change" factor): copper from brakes [12] and aldehydes from engine emissions [13]. Note that photochemical processes [14] are an unlikely source for this factor, since urban background and roadside levels near free-flowing traffic were much lower than in-vehicle levels [15]. The source of the high sulfur loading is unclear. Diesel combustion of accelerating trucks would be a plausible source candidate. However, sulfur is ubiquitous on secondary urban aerosols. It was the most concentrated element on PM_2.5 _in the study [15]. This prevents the identification of local sources with sulfur as a tracer. A cautious interpretation might be that factor 4 reflects particles from speed-changing traffic mixed with secondary urban particles; a mixture expected on roads in an urban-sprawl area like Raleigh.

Model B proposes only three sources. However, they correspond in principle to the sources from Model A, except that the factors " crustal" and "steel wear" seem to be collapsed into a single source factor "road surface". This notion is supported by the good correlation between the corresponding factors.

The average PM_2.5 _concentration of ca. 23 μg/m^3 ^inside the vehicles was at a moderate level compared to the 24-hour National Ambient Air Quality Standard for PM_2.5 _of 65 μg/m^3^. The two methods used to measure PM_2.5 _were highly correlated. The differences of their correlations to the components (Table 1) and to the source factors (Tables 2) reflect the fact that two different methods were used to assess the particle mass [15]: PM_2.5Lightscatter _reflects mostly accumulation mode particles (0.2 to 2 μm), whereas PM_2.5Mass _includes some coarse dust including fine sand.

The "speed-change" factor (Models A and B) was significantly associated with increased percentage of neutrophil leucocytes in the circulating blood, with decreased percentage of lymphocytes, and with changes in markers of endothelial activation and hemostasis. Endothelial cells are a major storage site for von Willebrand factor [16], and plasma levels of vWF serve as markers for endothelial activation [17]. Protein C is an antithrombotic agent, it is activated on the endothelium and reduced in the blood after inflammatory stimulation due to protein C consumption [18]. Consequently, endothelial cells may be involved in both inflammatory and coagulatory responses to traffic particles. Blood urea nitrogen was also associated with the "speed-change" factor. This finding would be consistent with the postulated inflammation since blood urea nitrogen increases several hours after an inflammatory stimulus (pig model) [19]. Blood urea nitrogen and vWF lost significance when controlled for all potential confounders together, although the effect estimates were not much changed. It should be noted that including this many confounders into a model with a relatively small number of samples reduces the strength of the statistics considerably.

The "speed-change" factor was significantly associated with changes in MCV (independent of osmolality) and similar to the association for PM_2.5Lightscatter _with MCV reported earlier [11]. The present analysis suggests that particles originating from speed-changing traffic are an important source of this association with circulating red blood cell mean volume (while other red blood cell indices were not affected). This is consistent with in-vitro blood experiments, where high concentrations of particles caused dose-dependent hemolysis, which was explained by oxidative damage to the membranes [20]. Note that MCV increases with increasing doses of hemolytic chemicals [21]. Future studies might answer the question whether particle-induced oxidative stress caused the association observed between MCV and the "speed-change" source factor.

The heart beat interval MCL increased in association with the "crustal" factor and the "speed-change" factor. Additionally, the "speed-change" factor was associated with significant increases in heart rate variability (SDNN and PNN50) and frequency of supraventricular ectopic beats. This cardiac response suggests increased vagal tone mostly in response to "speed-change" traffic particles. Fluctuations in autonomic tone have been associated with the triggering of atrial arrhythmias [22]. Such fluctuations might also help explain the reported association between air pollution exposure and increases in arrhythmias in patients with an implanted cardioverter defibrillator [6].

The concentrations of particles and components in this study were low. Direct systemic effects seem therefore unlikely. However, the proposed endothelial activation could provide a link to pathological processes and the associated increase in cardiovascular morbidity and mortality [7], as follows: Once particles are deposited on the surfaces of the airways or alveoli, toxic products can quickly leach out or be produced on the surface of the particles. Given the small volume of surface liquid, this can result in high local concentrations. Copper and other transition metals can cause oxidative stress [23] and have been associated with inflammatory lung injury in human subjects [24] as well as airway epithelial cell injury in vitro [25]. This oxidative stress might induce responses in the adjacent cells. In the alveolar region, the distance to the capillary endothelium is about 100 nanometers. Liberation of pro-thrombotic and pro-inflammatory mediators are well-described consequences of oxidative stress to endothelial and other cells [26]. Inflammatory stimuli also might induce a vagal response [27]. The components copper, sulfur and aldehydes dominated the "speed-change" factor. They seem to merit further attention in future targeted studies on particle toxicology.

Surprisingly, the "steel wear" factor of Model A was not associated to any inflammatory markers, although metal content of particles has been reported to be associated with inflammatory processes [24,25,28]. It would be interesting to study such wear particles with regard to size and solubility of metals.

One limitation of this study is the fact that only the association between the mean exposure during the evening shift and the response on the following morning was studied. This design ensured that potential diurnal variations of exposure and health parameters could not mimic a dose-response association, and that the exposure inside the cars was followed by a long unexposed resting period. However, it cannot be excluded that exposures and follow-up at other times of the day could have resulted in different dose-response estimates. Another limitation is the study population, since the troopers were a homogenous group of young, healthy, non-smoking people in excellent physical condition. Consequently, it is possible that the relative response such as the %-increase of inflammatory blood components or ectopic heart beats might be different in the troopers as compared to what could be expected in the general population or in individuals with elevated cardiovascular risks that have higher baseline levels. A final limitation is that the source factor "speed-changing" traffic does not represent a single source but rather a combination of closely related sources such as break wear and engine exhaust products. Answering the question, which of these sub-sources was causing the effects, would require a larger number of subjects or targeted toxicological studies.

## Conclusions

Fine particulate matter from vehicular traffic may activate one or more signaling pathways that cause pro-inflammatory, pro-thrombotic and hemolytic responses in healthy young men. The changes in the heart rate variability suggest an increased parasympathetic input to the heart with an associated increase in arrhythmic events, possibly in response to mild lung inflammation. These findings suggest the hypothesis that pollutants emitted during speed-changing traffic conditions negatively impact the health risks of professional or otherwise frequent vehicle drivers and passengers, or other people exposed to these particles. A long-term cardiovascular risk to the troopers can not be excluded, especially when considering the reported increase in myocardial infarction among professional drivers [29] and the increase in mortality among people living near major roadways [9]. These findings might be helpful for designing targeted studies in the future that investigate causative pathways for health effects of PM_2.5_.

## Methods

The study was conducted in fall 2001 in Wake County, North Carolina, USA. The Institutional Review Board of the UNC School of Medicine approved the study. All subjects gave informed written consent. Data from nine non-smoking male Highway Patrol troopers were analyzed. Each was monitored from Monday to Thursday while working the 3 PM to midnight shift. The troopers refrained from alcohol, caffeine and any medication from 24 hours before the start until the end of their participation. Each patrol car was equipped with air quality monitors to measure their exposure during the shift as described earlier [15]. Particle mass was assessed by two methods: PM_2.5Mass _by weighing filters; and PM_2.5Lightscatter _based on lightscattering. "Aldehydes" refers to the sum of formaldehyde, acetaldehyde, acrolein, propionaldehyde, crotonaldehyde, n-butyraldehyde, benzaldehyde, valeraldehyde, tolualdehyde, hexanaldehyde, and 2,5-dimethylbenzaldehyde.

Health parameters were assessed by ambulatory electrocardiography during the work shift and the subsequent sleep phase, and by analyses of peripheral blood samples drawn 15 hours after completion of the shift as described earlier [11]. Heart rate variability (HRV) measures in the time and frequency domain were calculated for resting periods before and after the shift, and in the morning after awakening. For the analysis presented, only data from the morning resting period were used. Parameters included the mean cycle length of normal R-R intervals (MCL), the standard deviation of normal R-R intervals (SDNN) and the percentage of normal R-R interval differences greater than 50 msec (PNN50), low frequency (0.04 to 0.15 Hz), high-frequency power (0.15 to 0.40 Hz) and the ratio of low to high frequency power. The number of ventricular and supraventricular ectopic beats were counted during the shift and the contiguous night.

Blood was collected from an antecubital vein and analyzed [11]. The analyses included uric acid, blood urea nitrogen, gamma glutamyl transpeptidase, white blood cell count, red blood cell count, hematocrit, hemoglobin, mean red blood cell volume (MCV), neutrophils (count and %), lymphocytes (count and %), C-reactive protein, plasminogen, plasminogen activator inhibitor type 1, von Willebrand factor (vWF), endothelin-1, protein C, and interleukin-6.

### Statistical methods

Spearman correlations were calculated using SYSTAT 10 (Systat Software Inc., Richmond, CA), all other statistics using S-Plus 6.1 for Windows (Mathsoft Inc., Cambridge, MA).

For classification of the exposure by potential sources, a principal factor analysis (factanal procedure) with variance-maximizing rotation [30] was conducted after controlling for outliers and missing data. One silicon value measured inside a patrol car was an outlier, possibly due to a grain of sand. This value was replaced by an estimate based on the aluminum level (Al was highly correlated to Si). Missing data (3 values of benzene and 2 of aldehydes) were replaced by the mean of the component concerned. For data below the propagated detection limit, machine-readouts were used. Factors with sum of squares of factor loadings larger than one were retained. The number of exposure variables included was limited to obtain stable results with this relatively small number of individual samples: only variables with a clear association to PM_2.5 _and with reasonable data quality were used. In a first model ("Model A") PM_2.5_-components, that were significantly correlated to either PM_2.5Mass _or PM_2.5Lightscatter _(Spearman Rho > 0.3), and gaseous co-pollutants, that were strongly and significantly correlated (Rho > 0.5) were included in the source factor analysis if at least 75% of the data were above reporting limit. A second model ("Model B") was calculated to assess the robustness of the source factor modeling and the associated health effects. Model B excluded PM_2.5_-components from the analysis with large uncertainties (Cr, Se and W with over 50% of data less than 3 sigma above background noise) or with weak correlation to PM_2.5 _(Ca).

Mixed effects regression models with restricted maximum-likelihood estimation, exposure factors as fixed effects and an unconstrained variance-covariance structure with subjects as grouping factors were used to investigate the associations between exposure and health endpoints [11,31]. Potential confounders were controlled for by including them into the models. Model testing included alternative, constrained variance-covariance structures (first-order autoregressive as well as linear and exponential spatial designs), and adding exposure factors to the random effects structure. None of these attempts improved the overall quality of the models judged by the Akaike Information Criterion, analysis of variance and residual analysis (distribution and autocorrelation). Consequently, only results from unconstrained models are reported.

## List of abbreviations

HRV heart rate variability

MCL mean cycle length of normal R-R intervals

MCV mean red blood cell volume

PNN50 percentage of normal R-R interval differences greater than 50 msec

SDNN standard deviation of normal R-R intervals

VWF von Willebrand factor

## Competing interests

The authors declare that they have no competing interests.

## Authors' contributions

MR conceived of and lead the study, collected the samples, performed the statistical analysis and drafted the manuscript. RBD supervised the analyses of blood components and elemental PM_2.5_-components. TRG supervised the on-site health assessment and the coordination of the troopers. MCH analyzed the ambulatory electrocardiograms and calculated the HRV statistics. PAB participated in the study management and in the layout of the manuscript. RWW supervised the assessment of air pollutants. WEC evaluated the volunteering troopers, and supervised the heart data analysis. All authors participated in the study design, and reviewed and approved the final manuscript.
